# Effects of exogenous lipids and cold acclimation on lycopene production and fatty acid composition in *Blakeslea trispora*

**DOI:** 10.1186/s13568-019-0891-5

**Published:** 2019-10-11

**Authors:** Feng Lingran, Wang Qiang, Yu Xiaobin, Fred Kwame

**Affiliations:** 1 College of Life Sciences, Henan Normal University, Xinxiang, 453007 China; 2 The Key Laboratory of Industrial Biotechnology, Ministry of Education, and School of Biotechnology, Jiangnan University, Wuxi, 214122 China

**Keywords:** Lycopene, Cold acclimation, Exogenous lipids, *Blakeslea trispora*

## Abstract

Exogenous lipids serving as stimulators to improve lycopene production in *Blakeslea trispora* have been widely reported. However, the selection basis of exogenous lipids and their effects on intracellular lipids are not very clear. In this study, five plant oils with different fatty acid compositions were selected to investigate their effects on lycopene production, fatty acid composition and the desaturation degree of intracellular lipids. Among the oils, soybean oil, with a fatty acid composition similar to that of mycelium, exhibited the best stimulating effect on lycopene formation (improvement of 82.1%). The plant oils enhanced the total content of intracellular lipids and the desaturation degree of reserve lipids due to the alteration of fatty acid composition, especially in neutral lipids. Lycopene production was increased with the improved desaturation degree of intracellular lipids, which may be attributed to the enhancement of storage capacity for lycopene in storage lipid, thus reducing the feedback regulation of free lycopene. In addition, the increase of the desaturation degree of reserve lipids through temperature-changing fermentation also enhanced lycopene production. The present study could serve as a basis for a better understanding of the relationship between the fatty acid composition of reserve lipids and lycopene production.

## Introduction

Lycopene, a member of the carotenoids, has been widely applied in the pharmaceutical, feed, and food industries (Feofilova et al. 2006; Nelis and Leenheer 1991). Increasing evidence supports the role of lycopene against some diseases, such as cardiovascular diseases and prostate cancer (Khachik et al. 2002; Story et al. 2010). Lycopene can be synthesized by chemical pathways, plants and microorganisms. Compared with chemical synthetic lycopene, natural-origin lycopene extracted from plants (mainly tomato) and microorganisms (mainly *B. trispora*) has a broader market due to the increasing consumer demand for “all natural” products, although synthetic and natural lycopene have identical bioavailability in humans (Hoppe et al. 2003; Papaioannou et al. 2016). The main advantages of natural lycopene are more eco-friendly and safe to human compared with synthetic lycopene (Martínez-Cámara et al. 2018). Natural lycopene is approved for coloring matters within European Union and FDA, but chemical synthetic lycopene is not permitted. Moreover, the consumer perception that “natural is good” has been motivating the increase of natural lycopene markets. Although plant-origin lycopene still prevails in the current lycopene market, microbial lycopene has been commercialized by some companies employing the fungus *B. trispora*, such as Vitatene (Leon, Spain) and the North China Pharmaceutical Group (Shijiazhuang, China). Compared with plants, microorganisms have the advantages of a rapid growth rate, controllable conditions, and a high purity and percentage of lycopene in cells (López-Nieto et al. 2004). *B. trispora*, a zygomycota fungus with two mating types (termed ‘plus’ and ‘minus’), occupies a prominent place among microbial producers of lycopene. Lycopene is an intermediate of the carotenoid biosynthetic pathway in *B. trispora* strains. To obtain lycopene, either a cyclase inhibitor, i.e., 2-methylimidazole (Pegklidou et al. 2008), or genetic means is required to prevent the formation of β-carotene and promote the accumulation of lycopene. Similar to plant origins, lycopene configurations in *B. trispora* mainly consist of all-*trans* forms (> 90%), although lycopene in vitro can isomerize to the mono or poly-*cis* form under heating or light (Authority 2005; Shi 2000).

The low yield of the final product is one of the main limitations of lycopene production by *B. trispora*. Numerous studies revealed that mutation breeding (Mehta et al. 2003; Rodriguez et al. 2009), various stimulants (Bhosale 2004; Shi et al. 2012; Wang et al. 2012), and process optimization (Mantzouridou et al. 2005, 2002; Mantzouridou and Naziri 2017) have been successfully used for enhancing lycopene production. Growth and lycopene synthesis are known to be stimulated by vegetable oils with high contents of linoleic and especially linolenic acids (Nanou and Roukas 2016; Sevgili and Erkmen 2019). The addition of plant oils improved the content of cellular unsaturated triacylglycerol in *B. trispora* compared with that obtained using glucose as the sole carbon source (Mantzouridou and Tsimidou 2007). Theoretically, exogenous lipids can be used for (a) synthesizing intracellular compounds and obtaining energy and (b) the biosynthesis of both lycopene and cellular lipids because they use acetyl-CoA (resulting from lipid degradation) as a common precursor substance. The catabolism of lipids promotes glucose towards the hexose monophosphate pathway (HMP) due to intracellular energy excess, hence increasing the production of NADPH. Thus, cellular lipid and lycopene synthesis can be stimulated because the biosynthesis and desaturases of both carotenoids and aliphatic chains require NADPH as co-factors (Beyer et al. 1994; Garba et al. 2017; Miziorko 2011). However, the effects exogenous lipids on intracellular lipids and the relationship between cellular fatty acid composition and lycopene production are not very clear.

Several modes of change of cellular fatty acids at varying environmental temperatures have been found in fungi, yeasts and bacteria, such as the degree of fatty acid unsaturation, fatty acid chain length, and cellular fatty acid content. The main change reflects the degree of unsaturation of fatty acyl chains through the control of fatty acid desaturation both at the level of transcription and post-transcriptional regulation (Dyer et al. 2001; Gargano et al. 1995; Vigh et al. 2005). Remodeling of membrane composition and functionality to maintain proper cellular function is widely accepted and termed homeoviscous adaptation (Cossins 1994). In addition to phospholipids, *B. trispora* also reserves large amounts of neutral lipids in lipid bodies where lipophilic carotenoids are deposited. It has been shown that hypothermic conditions can result in (a) an increase in the degree of desaturation of fatty acids in the lipid bilayer and (b) changes in the qualitative and quantitative composition of neutral lipids (Feofilova et al. 2000). Feofilova et al. (2005) showed that the degree of desaturation of fatty acids of both (+) and (−) strains of *B. trispora* increased in the lipid bilayer, although the (−) strain lacks linolenic acid.

Plant oils are known to improve the lycopene production and biomass of *B. trispora*. However, their effects on intracellular fatty acid composition and the selection basis of exogenous plant oils are not very clear. In this work, the lipid composition and lycopene production of *B. trispora* in response to different exogenous plant oils and temperature variation were investigated. The aims were to reveal the effects of several common plant oils on intracellular lipids and the relationship between cellular fatty acid composition and lycopene production. Based on these findings, lycopene production could be improved by altering the intracellular fatty acid composition of *B. trispora*, such as by adding suitable plant oils, changing the fermentation temperature, and adjusting other culture conditions.

## Materials and methods

### Microorganisms and culture conditions

The microorganisms used in this study were *B. trispora* NRRL 2895 mating type (+) and *B. trispora* I5 mating type (−). The strain I5, which presents a super yellow colony color and lovastatin resistance, was a high-yield lycopene mutant of *B. trispora* NRRL 2896 mating type (−) (Wang et al. 2013). The strains were grown on potato dextrose agar medium at 25 °C for 4 days and thereafter subcultured every 30 days. The spores obtained were suspended in 10 mL of sterile water to prepare the inocula. Spore suspensions containing 2.0 × 10^5^ and 1.0 × 10^6^ spores/mL of the strains NRRL 2895 and I5 was transferred to 250-mL conical flasks, each containing 50 mL of seed medium. The flasks were cultivated on a rotary shaker with an agitation rate of 200 rpm at 25 °C for 48 h. The seed medium (g/L) contained: corn starch 30, soybean meal 50, KH_2_PO_4_ 1.5, MgSO_4_·7H_2_O 0.5 and thiamine·HCl 0.002, and the pH was adjusted to 6.5. All medium components were sterilized at 121 °C for 20 min.

### Fermentation conditions

Fermentation was carried out in 250-mL flasks with 25 mL of media (three replicas of each treatment) and 10% (v/v) inoculum containing a 1:5 (v/v) mixture of each seed broth grown separately. The fermentation medium (g/L) contained: corn starch 50, soybean meal 25, cottonseed oil 40, KH_2_PO_4_ 1.5, MgSO_4_·7H_2_O 0.5 and thiamine·HCl 0.002, and the pH was adjusted to 6.5. All medium components were sterilized at 121 °C for 20 min. The flasks were cultivated on a rotary shaker with an agitation rate of 200 rpm at 25 °C. A cyclase inhibitor, i.e., 2-methylimidazole, was added at a concentration of 0.3 g/L after 48 h of fermentation (Pegklidou et al. 2008). Cultures were maintained for 120 h, and then the cells were harvested to determine the dry cell weight and lycopene content.

#### Temperature-changing fermentation

To evaluate the effect of different culture temperature on the growth of *B. trispora*, the inoculated flasks were cultivated at different constant temperature that as follows: 16, 18, 20, 22, 24, 26, and 28 °C. Cultures were maintained for 120 h, and then the cells were harvested to determine the dry cell weight.

To obtain the time course of lycopene concentration and dry biomass by *B. trispora* under constant temperature and varying temperature, the experiments were designed as follows. 72 inoculated flasks were divided into two groups: constant temperature and varying temperature. During constant-temperature fermentation, the temperature was maintained at 25 °C until the end. During temperature-changing fermentation, the culture process was divided into three phases: phase I for the first 84 h, phase II for the following 24 h, and phase III for the last 36 h. The temperatures were maintained at 25 °C for phase I and phase III, and 20 °C for phase II. Every 12 h interval, three flasks of each group were taken out to determine the lycopene concentration and dry cell weight. In addition, the samples collected from the varying temperature group at 72, 96 and 120 h were simultaneously used to determine the fatty acid composition of intracellular lipid of *B. trispora*.

### Lipid and fatty acid analysis

At appropriate time intervals, a 10-mL sample was collected from the culture broth and then centrifuged at 10,000×*g* for 20 min. The sediment was disrupted by freezing in liquid nitrogen and subsequently thawing and disintegrating with quartz sand. Lipid was extracted by using a chloroform–methanol (2:1) mixture that was stirred on a magnetic mixer. Lipid extracts were separated on thin-layer chromatography plates (20 × 20 cm) coated with silica gel 60, using solvents with different degrees of polarity (Nichols 1963). Neutral lipids (NL) were separated using the solvent system hexane-ether-acetic acid (85:15:1). Phospholipids (PL) were separated using two solvent systems successively in the same direction: hexane–ether–acetic acid (85:15:1) and chloroform–methanol–acetic acid–water (25:15:4:2). Glycolipids (GL) were separated using the solvent system chloroform–methanol–water (65:15:2). The lipid fractions containing NL, PL, GL were scraped off the plates for fatty acid analysis, respectively. Lipid classes were visualized by iodine vapor and identified by comparison of their R_f_ values with those of known standards (Sigma). Individual lipid classes were relatively quantified by a densitometer. Fatty acid methyl esters (FAMEs) were prepared as described by Lepage and Roy (1984) and then analyzed using a gas chromatography mass spectrometry (Thermo Scientific TQ 8000, USA) equipped with a Supelco SPB-50 capillary column (30 m × 0.25 mm, 0.25 μm). The column temperature was programmed using the gradient mode starting at 110 °C maintained for 3 min, then gradually increasing to 220 °C at a rate of 20 °C min^−1^ and maintaining the latter temperature for 25 min. The temperature of the injector and detector were maintained at 250 and 260 °C, respectively. Samples (1 μL) were injected with a split ratio of 50:1 by the autoinjector. Helium was used as a carrier gas. The ion source and ion source surface temperatures were set to 200 °C and 250 °C, respectively. Electron impact ionization (70 eV) in scan mode (m/z 60–600) at a rate of 20 scans/s was used. Mass spectra of all detected compounds were compared with spectra in the NIST library 2.0 (2008 version) and the in-house mass spectra library database established by Umea Plant Science Center. Individual fatty acids were identified by comparing their retention times with those of known FAME standards (Sigma). Nonadecanoic acid (C19:0) was used as an internal standard. Fatty acid amounts were relatively quantified by calculating their chromatographic peak areas.

### Extraction and analysis of lycopene

After 120 h, a 10-mL sample was collected from culture broth and then centrifuged at 10,000×*g* for 20 min. The sediment was washed with distilled water and recentrifuged (triple). Dry biomass weight was determined after drying at 105 °C overnight. To measure lycopene content, the sediment was dried in a vacuum drier at 40 °C and then crushed using mortar and pestle, followed by extraction of lycopene with petroleum ether. Repeated extractions of lycopene were carried out with the above solvent until the residue became colorless. Lycopene produced was analyzed by high-performance liquid chromatography (HPLC). The HPLC was equipped with a ZORBAX Eclipse Plus C18 column (250 mm × 4.6 mm) at 30 °C. The mobile phase was acetonitrile: acetone (60:40, v/v) at a flow rate of 1 mL/min. The absorption of lycopene was measured at 470 nm. Lycopene was identified by comparing with the retention time of lycopene standard (Sigma), and quantitative analysis was performed by the single-point calibration method.

### Statistical analysis

The reported data are mean values of three independent experiments. Statistical differences between different treatments (groups) were determined by analysis of variance (ANOVA) and Tukey’s post hoc test. Results were considered significant when differences had values of p < 0.05. Pearson’s correlation was used to analyze the relationships between the solubility of lycopene and the desaturation degree of plant oils. All statistical analyses were performed with SPSS 18.0 (SPSS Inc., Chicago, Illinois).

## Results

### Effect of exogenous lipids on dry biomass and lycopene production

**Fig. 1:**
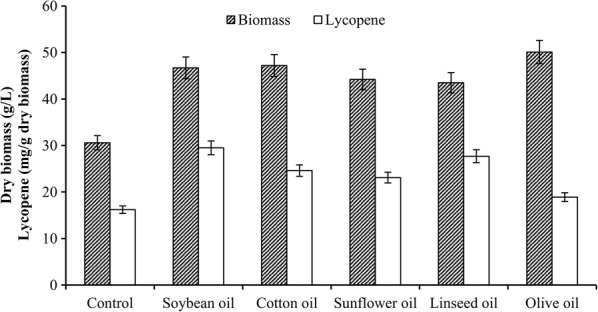
Effect of different plant oils on dry biomass and lycopene production by *B. trispora*. Error bars indicate SEs of mean values. Black-striped bars, biomass; off-white-colored bars, lycopene

According to the available literature, the lipids of *B. trispora* grown in media with glucose or corn flour predominantly contained palmitic (C_16:0_), stearic (C_18:0_), oleic (C_18:1_), linoleic (C_18:2_), and linolenic (C_18:3_) fatty acids (Tereshina et al. 2005, 2010; Vereschagina et al. 2010). When corn starch was used as the main carbon source, the effects of the oils used in this study on total lipids, dry biomass, and lycopene production are shown in Fig. 1. In the presence of oils, these three parameters were increased relative to those without oil. Soybean oil was more effective in stimulating lycopene formation (40.1 mg/g) than other oils (p < 0.05, ANOVA analysis and Tukey’s post hoc test). The highest volumetric production (1.3 g/L) was obtained in the presence of soybean oil (p < 0.01, ANOVA analysis and Tukey’s post hoc test). The fatty acid compositions of the oils were determined by assessing fatty acid profiles (Table 1). These plant oils largely contained saturated palmitic and stearic fatty acids and unsaturated oleic, linoleic, and linolenic fatty acids. Among these oils, the highest contents of palmitic, oleic, linoleic, and linolenic fatty acids were found in cotton, olive, soybean, and linseed oils, respectively (p < 0.05, ANOVA analysis and Tukey’s post hoc test). The contents of stearic acid in these oils were not significantly different (p > 0.05, ANOVA analysis and Tukey’s post hoc test).

**Table 1:** Effect of plant oils on the fatty acid composition of intracellular lipids of *B. trispora*

	Control			Soybean oil			Olive oil			Linseed oil		
	NL	PL	GL	NL	PL	GL	NL	PL	GL	NL	PL	GL
C_14:0_	2.9^a^	2.5	10.5	3.3	1.0	8.6	3.5	1.8	9.7	3.0	2.1	9.1
C_15:0_	–	–	–	–	0.4	–	–	–	0.6	–	–	0.5
C_16:0_	21.3	22.8	18.9	10.7	15.1	12.9	12.4	18.8	13.5	11.5	21.0	14.5
C_16:1_	0.3	–	0.3	0.5	–	0.5	0.4	–	0.6	–	–	0.4
C_17:0_	–	3.2	–	1.0	1.7	–	–	1.5	–	–	1.5	–
C_18:0_	15.1	5.0	19.5	6.2	3.3	14.8	8.0	3.8	12.7	8.7	4.7	14.3
C_18:1_	21.9	21.2	21.1	25.1	20.5	24.5	56.3	29.7	30.5	20.5	19.7	23.4
C_18:2_	34.3	44.8	27.2	46.5	47.3	33.0	12.4	42.9	26.3	26.3	45.9	30.2
C_18:3_	1.9	0.5	–	4.1	0.7	–	4.0	0.7	–	18.5	4.7	3.5
C_20:0_	0.8	–	–	0.5	0.4	0.6	0.7	–	0.5	0.7	–	0.4
C_20:1_	1.0	–	1.8	0.4	–	3.5	0.6	–	3.0	–	–	0.5
C_20:2_	–	–	–	–	–	0.4	1.0	0.8	1.7	0.5	0.4	1.5
C_20:3_	–	–	–	–	–	–	–	–	–	–	–	0.4
C_20:4_	–	–	0.4	–	–	0.3	–	–	0.5	–	–	0.3
C_22:0_	0.5	–	0.3	0.7	–	0.5	0.7	–	–	–	–	0.5
C_22:1_	–	–	–	–	–	0.4	–	–	0.4	–	–	0.5
Desaturation degree	0.98	1.12	0.79	1.31	1.17	0.97	0.96	1.19	0.93	1.30	1.26	1.01

^a^All value mean the relative content of fatty acids, % of lipid fraction

### Effect of plant oils on fatty acid composition of cellular lipids

To further investigate the influence of oil on lycopene synthesis, the fatty acid compositions of the NL, PL, and GL of the mycelium in the presence of different oils were assayed. Based on the composition analysis of plant oils, unsaturated fatty acids showed a positive influence on lycopene production. Three plant oils (soybean, linseed, and olive oils) with significantly different degrees of unsaturation were chosen for further analysis (p < 0.01). Meanwhile, their effects on lycopene production also showed significant differences (p < 0.01). The results are shown in Table 1. The overall trend of fatty acid composition in the presence of oils was an increase in unsaturated fatty acids against a decrease in saturated fatty acids relative to the compositions without oil. This resulted in an increase in the desaturation degree of fatty acids of the NL, PL, and GL of the mycelium. Unlike the strong influence of soybean and linseed oils on the desaturation degree of fatty acids, olive oil was relatively weak. This may be attributed to the difference in the fatty acid compositions of these three oils. The relative contents of the lipid fractions and the total lipid contents in the mycelium in the presence of different oils are shown in Table 2. Both NL and total lipid were increased in the presence of oils compared with those without oil. The percentage increase of NL in lipid fractions was not measured against the background of the decreased absolute contents of PL and GL. This is due to the significant increase in the absolute content of NL compared with the slight increase in PL and GL.

**Table 2:** Effect of culture temperature on fatty acid composition of intracellular lipid of *B. trispora*

	Phase I^a^			Phase II			Phase III		
	NL	PL	GL	NL	PL	GL	NL	PL	GL
C_14:0_	3.5^b^	2.5	8.7	1.2	0.5	6.1	3.0	2.7	7.2
C_15:0_	–	–	–	–	–	0.5	–	0.3	0.3
C_16:0_	15.4	20.8	18.5	3.0	7.8	7.2	7.2	18.4	17.1
C_16:1_	0.5	–	0.3	0.6	–	–	0.7	–	–
C_17:0_	1.2	3.2	–	0.8	0.4	0.4	0.8	3.0	0.7
C_18:0_	18.2	4.7	11.2	9.0	2.5	9.1	12.8	5.8	11.1
C_18:1_	23.8	21.5	24.0	32.2	33.2	31.2	24.5	20.5	26.4
C_18:2_	33.2	46.8	33.6	48.4	55.2	37.9	43.2	47.6	35.4
C_18:3_	2.4	0.5	–	3.0	4.6	–	6.3	1.5	0.5
C_20:0_	0.5	–	0.3	0.7	0.4	0.5	0.5	0.2	0.3
C_20:1_	0.4	–	3.2	–	–	4.6	–	–	–
C_20:2_	–	–	0.4	0.3	–	–	0.3	–	–
C_20:3_	–	–	0.3	–	–	0.2	–	–	0.4
C_20:4_	–	–	0.6	–	0.4	1.1	–	–	0.3
C_22:0_	0.9	–	0.5	0.8	–	0.3	0.7	–	–
C_22:1_	–	–	0.4	–	–	0.9	–	–	0.3
Desaturation degree	0.98	1.17	0.99	1.39	1.59	1.18	1.31	1.20	1.01

^a^Sampling time of Phase I, Phase II and Phase III were 72, 96, and 120 h, respectively

^b^All value mean the relative content of fatty acids, % of lipid fraction

### Solubility of lycopene in different plant oils

**Fig. 2:**
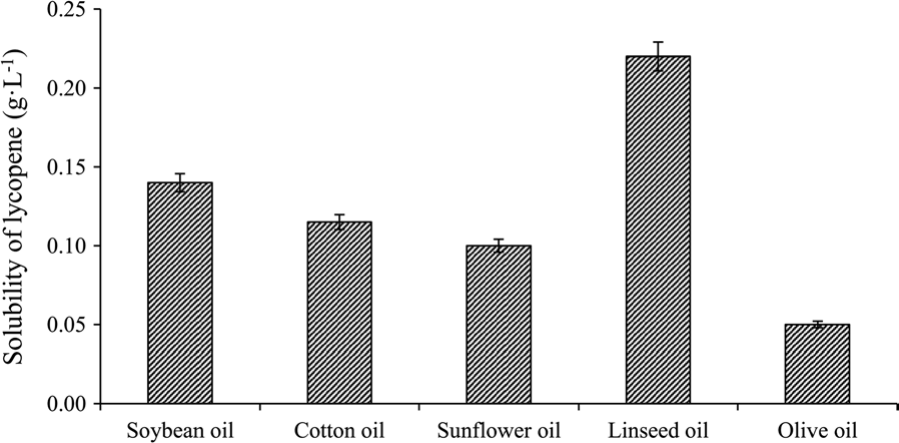
Solubility of lycopene in different plant oils. Error bars indicate SEs of mean values

The fatty acid compositions of intracellular lipid have some similarities with that of plant oils. Therefore, to explore the influence of the fatty acid composition of intracellular lipid on the solubility of lycopene in mycelium, the solubilities of lycopene in different plant oils were determined. As Fig. 2 shows, linseed oil exhibited the highest solubility value, which was two times higher than secondary soybean oil. Olive oil showed the lowest solubility value. In addition, there was a positive correlation between the solubility of lycopene and the desaturation degree of plant oils (pearson correlation, R^2^ = 0.972, p < 0.01).

### Effects of temperature variation on dry biomass and lycopene production

**Fig. 3:**
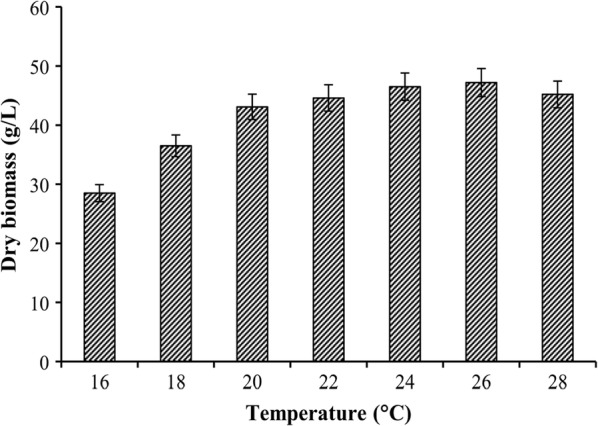
Effect of different culture temperature on dry biomass by *B. trispora*. Error bars indicate SEs of mean values

**Fig. 4:**
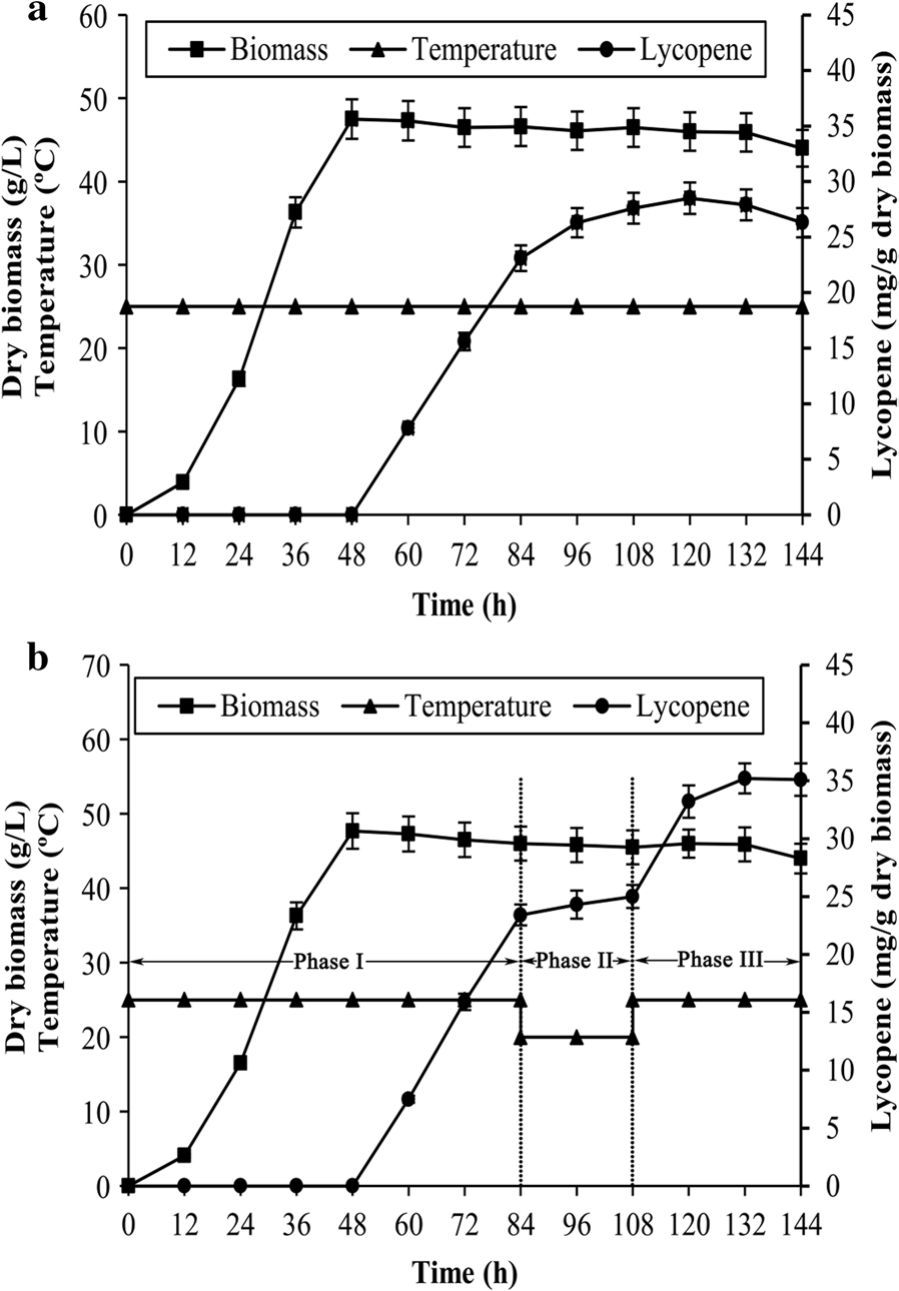
Time course of lycopene concentration and dry biomass by *B. trispora* under **a** constant temperature and **b** varying temperature. Error bars indicate SEs of mean values. Solid square, dry biomass; solid triangle, temperature; solid circles, lycopene concentration

It has been shown that cold acclimation can alter fatty acid composition in intracellular lipids, especially the degree of unsaturation of fatty acyl chains. Therefore, the effects of temperature variation on lycopene production and fatty acid composition were investigated, with the aim of providing support for the correlation between lycopene production and the desaturation degree of intracellular lipids. As shown in Fig. 3, the growth of *B. trispora* was obviously restricted when the culture temperature was below 20 °C. Hence, the cold stress temperature was chosen as 20 °C. The time courses of dry biomass and lycopene production under a suitable growth temperature are shown in Fig. 4a. The biomass increased rapidly at 12 h, reached the maximum value at 48 h, and remained almost constant afterwards. Lycopene production increased rapidly after adding cyclase inhibitor at 48 h and increased slowly after 84 h. Figure 4b shows the changes of the dry biomass and lycopene production with fermentation time under temperature variation. From Fig. 4a, the time of culture temperature adjustment to 20 °C ranged from 84 to 108 h. The change in biomass under this variation was similar to that under constant temperature. Although lycopene concentration increased slightly under cold stress, it increased rapidly when the culture temperature was set to 25 °C again. The final lycopene concentration reached 35.2 ± 1.1 mg/g dry biomass under varying culture temperatures, which was 24% higher than that under constant temperature. The optimum temperature for lycopene production is not 20 °C, since the lycopene concentration remained almost constant at this temperature.

### Changes in fatty acid composition in cellular lipids under temperature variation

The fermentation process was segmented into three phases based on temperature change. The fatty acid compositions of NL, PL, and GL during these three phases are shown in Table 3. When fermentation was carried out to phase two, the fatty acid composition exhibited considerable changes due to the influence of the hypothermic conditions. The main modulations involved the increase of unsaturated oleic and linoleic fatty acids against the background of the decrease in saturated stearic and palmitic fatty acids. During phase three, the fatty acid composition of PL and GL almost returned to that in phase one. Surprisingly, the fatty acid composition of NL remained similar to that in phase two; i.e., NL contained high contents of unsaturated oleic and linoleic fatty acids, although culture temperature was recovered. Hence, the desaturated degree of fatty acids of NL increased and persisted until phase three.

**Table 3:** Effect of plant oils on the composition and content of intracellular lipid and NADPH concentration by *B. trispora*

	Lipid fractions, % of total lipids			Total content of lipids, % of dry biomass	NADPH (nmol/mg protein)
	NL	PL	GL		
Control	80.4	15.0	4.6	15.3 ± 0.6	0.42 ± 0.01
Soybean oil	92.5	5.6	1.9	45.3 ± 2.1	1.1 ± 0.01
Olive oil	92.5	5.8	1.7	43.1 ± 2.3	0.93 ± 0.02
Linseed oil	89.0	8.5	2.5	31.2 ± 1.4	1.02 ± 0.01

Data of absolute content are expressed as the mean ± SD from three replications

## Discussion

Exogenous plant oils are hydrolyzed to fatty acids and glycerol by fungal exolipases. Fatty acids can (a) be oxidized to acetyl-CoA by β-oxidation and (b) be incorporated into reserve lipid structures, such as lipid bodies (Čertík et al. 1997). Acetyl-CoA not only provides energy for metabolism through the tricarboxylic acid cycle but also serves as a common precursor of carotenoids and lipids. Consequently, the increase of precursor stimulates the formation of downstream products. Lycopene is a highly unsaturated hydrocarbon that contains 11 conjugated and two unconjugated double bonds (Shi and Maguer 2000). The desaturation degree of lycopene is much higher than that of intracellular lipids or plant oils. Thus, the increase in the desaturation degree of intracellular lipids was favorable for the dissolution of lycopene. The direct incorporation of exogenous fatty acids, especially linoleic and linolenic acids, enhances the desaturation degree of the fatty acids of the acylglycerols, thus increasing the solubility of lycopene (Mantzouridou and Tsimidou 2007). Therefore, this promotes the storage of large amounts of lycopene in the lipid bodies, which mainly consist of acylglycerols (Danilova and Tereshina 2019; Walther and Farese Jr 2012). NADPH is an essential cofactor in both the elongation and desaturation of the carbon chain of lycopene. One mole of produced lycopene requires 16 mol of NADPH and 24 mol of ATP (data not shown). In oil-enriched substrates, intracellular energy is excessive, resulting from the catabolism of fatty acid-based substrates. Hence, more glucose is metabolized through the HMP pathway, thereby increasing the production of NADPH (Reshamwala and Modi 1985). This, together with the reduced demand for NADPH, ATP and carbon in fatty acid biosynthesis due to the direct incorporation of extracellular unsaturated aliphatic chains, results in the enhanced supply of NADPH, ATP and carbon for carotenoid synthesis.

Among the NL, PL, and GL, the fatty acids of the NL showed the most significant changes in response to different oils. In contrast, the fatty acids that prevailed in the oils were not significantly accumulated in the PL and GL of the mycelium. The GL acts as a recognition site for specific chemicals as well as helps to maintain the stability of the membrane (Schnaar 2004). PL is the major component of the cell membrane and can provide membrane fluidity and mechanical strength (Bloom et al. 1991). Owing to these special functions, the fatty acid components of the GL and PL were steady. NL has a role in energy and lipophilic substance storage (Athenstaedt 2010). Its content is large and variable. The highest fatty acid components of soybean, olive, and linseed oils are linoleic (C_18:2_), oleic (C_18:1_), and linolenic (C_18:3_) fatty acid, respectively. These acids significantly accumulated in the NL of the mycelium owing to the addition of these three oils. The desaturation degree of fatty acids of NL decreased slightly, although the absolute content of unsaturated oleic acid increased with olive oil addition. This is because the relative contents of linoleic, linolenic and other unsaturated acids decreased with the increase in the absolute content of oleic acid. Calculation of the desaturation degree was based on the relative contents of these fatty acids. The uptake of oils can not only enhance the content of NL of the mycelium but also increase the desaturation degree of fatty acids of the NL (Mantzouridou and Tsimidou 2007). Therefore, more lycopene can be deposited in storage lipid thus reducing the feedback inhibition of free lycopene. Among these oils, soybean oil exhibited the best stimulating effect on lycopene production. In addition to the above mechanism, soybean oil contains high levels of lecithin, a very effective emulsifier of lycopene that can facilitate its transfer from one enzyme to the next.

Lycopene is a secondary metabolite and not necessary for growth in *B. trispora*. Microbes reduce or even suspend unimportant secondary metabolism to maintain basic functions under cold stress (Feofilova 2003). Energy and building material used for lycopene biosynthesis may be reduced under cold stress, resulting in the deceleration of lycopene biosynthesis (phase II in Fig. 4b). But lycopene concentration increased rapidly after returning to normal culture temperature as expected (phase III). The reason for this phenomenon may be the change of lycopene solubility depending on the alteration of fatty acid composition in the reserve lipids. Hence, the changes in fatty acid composition in cellular lipids at varying temperatures were investigated. Microbes respond to temperature stress by changing the composition of the membrane and reserve lipids (Beney and Gervais 2001; Zhu et al. 2007). The changes that occur in lipid acyl chains mainly include saturation and desaturation, isomerization, and changes in the length of fatty acid carbon chains (Suutari and Laakso 1994; Wagenen et al. 2012). The data obtained enabled us to hypothesize that the changes in the fatty acid composition of NL are difficult to recover, for a time at least. The enhancement of lycopene under cold stress can be mainly attributed to the increase of the desaturation degree of NL. Combined with the influence of plant oils on lycopene production and the fatty acid composition of intracellular lipids, it can be concluded that the increase in the desaturation degree of intracellular lipids benefits the accumulation of lycopene in *B. trispora*.

The oils with fatty acid compositions similar to that of the fungus had more advantages in altering intracellular fatty acid composition and enhancing lycopene production relative to other oils. To our knowledge, this is the first study on the effect of cold acclimation on lycopene biosynthesis by *B. trispora*. Lycopene production were substantially improved just changing fermentation temperature, which may bring some commercial interests. In addition to cold acclimation, other conditions, such as oxygen concentration and pH, can also affect fatty acid composition and lipid content. Therefore, these conditions could be experimentally determined to enhance lycopene production in future studies.

## Data Availability

Not applicable

## Abbreviations

HMP: hexose monophosphate pathway
NL: neutral lipids
PL: phospholipids
GL: glycolipids
FAMEs: fatty acid methyl esters
HPLC: high-performance liquid chromatograph
